# Aflatoxicosis in Pekin duckling and the effects of treatments with lycopene and silymarin

**DOI:** 10.14202/vetworld.2021.788-793

**Published:** 2021-03-29

**Authors:** Sahar M. El-Sheshtawy, Amal F. El-Zoghby, Nesreen A. Shawky, Dalia H. Samak

**Affiliations:** 1 Toxicology Animal Health Research Institute, Tanta Branch, Agriculture Research Centre, Giza, Egypt; 2 Animal Health Research Institute, Tanta and Zagazig Branch, Agriculture Research Centre, Giza, Egypt; 3 Department of Veterinary Forensic Medicine and Toxicology, Faculty of Veterinary Medicine, Damanhour University, Egypt

**Keywords:** aflatoxins, aspergillus, creatinine, hepatic, kidney, lycopene, mycotoxicosis, oxidative stress, pekin duckling, residue, silymarin

## Abstract

**Background and Aim:**

Aflatoxins (AFs) are potent toxic metabolites produced from *Aspergillus* species. Whose existence in poultry ration leads to drastic economic losses, notably in duck, as the most susceptible poultry species. This study aimed to determine tissue residues of AFs, alterations in selected clinical chemistry variables in serum, mainly during the exposure period, and lycopene and silymarin’s possible roles as herbal treatments against aflatoxicosis in Pekin duckling.

**Materials and Methods:**

The study used one hundred and twenty one-day-old Pekin ducklings and classified them into four groups comprising 30 ducklings in each group. The control group (G1) ducklings were fed a mycotoxin-free ration, and G2 received a naturally contaminated ration with 30 ppb of AFs. G3 and G4 consumed contaminated rations with AFs with 30 ppb for 2 weeks and were treated with lycopene 100 mg/kg or silymarin 600 mg/kg/food, respectively, for 10 days. Serum activities of alanine transaminase and alkaline phosphatase (ALP), glutamyl transferase, ALP, total protein and albumin creatinine and uric acid concentrations, oxidant/antioxidant parameters (malondialdehyde [MDA], total antioxidant capacity (TAC), glutathione S-transferase (GST), and catalase [CAT]), and hepatic AFs residue were determined. Lycopene and silymarin were used for the treatment of aflatoxicosis for another 10 days.

**Results:**

Hepatic and kidney parameters were elevated in the AFs intoxicated group and reduced in the lycopene- and silymarin-treated groups. They had elevated MDA and AFs residues with decreased antioxidant parameters (TAC, GST, and CAT) in the AFs group. At the same time, treatment with lycopene or silymarin had reversed the action of AFs on MDA, elevated the hepatic residue, and improved antioxidant activity.

**Conclusion:**

Lycopene and silymarin, with their potent antioxidant activity, can be used to reverse the harmful effects of AFs on hepatic and kidney tissue.

## Introduction

Aflatoxins (AFs) come from fungi named *Aspergillus* species as secondary toxic metabolites [1]. Compared with broiler species, there is limited research on aflatoxicosis in ducks even though they are the most sensitive species to AFs. They have a higher bioactivation activity for AFs than any poultry species [2-4]. In 2010, the worldwide duck meat industry was $3.9 million, with more than 50,000 tons produced in the United States, and 95% of consumption represented Pekin ducks [5]. AFs have a deleterious effect on most organs and tissues of the body, ­especially the liver. The International Agency for Research on Cancer has identified AFs as a powerful toxic form and recorded as a category one carcinogen [6], which can suppress the metabolic enzymes, ultimately resulting in liver, kidney, and heart injury [7], and reproductive hypofunction [8]. Microsomal CYP_450_ enzymes are responsible for AFs’ bioactivities to a highly toxic metabolite named AFs-8,9-epoxide (AFBO) [9].

This toxic mycotoxin metabolite binds with protein and nucleic acid leading to a harmful effect on [10] fatty acid metabolism and enhanced cellular oxidative stress [11-14]. AFs present in the food may affect livestock production, and their residues in animal tissue such as the liver, gizzard, kidney, and eggs are a potential hazard for public health [15-17]. Management of aflatoxicosis without any adverse reaction is indeed a difficult task in medicine. At present, available aflatoxicosis drugs have negative impacts [18]. Lycopene is a natural carotenoid extracted from vegetables derived products such as tomatoes and red fruits (guava, watermelons, red peppers, pink grapefruit, and papayas). It can detoxify the toxic metabolites through its antioxidant, anticancer, anti-inflammatory, and anti-cardiovascular properties [19]. There is another plant that seems to have a heptatonic positive impact, which is called silymarin. Silymarin has several flavonolignans active constituents such as silychristin, isosilychristin, silibinin, silydianin, and isosilibinin and a flavonoid taxifolin. These active constituents can regenerate the liver cell by stabilizing the cell membranes, lipid peroxidation suppression and anti-inflammatory effect Furthermore, it has powerful antioxidant properties that regulate hepatoprotective activity in different animal models [20]. There is potential in the use of these herbal extracts in aflatoxicosis therapy.

This study aimed to determine tissue residues of AFs, alterations in selected clinical chemistry variables in serum, mainly during the exposure period, and lycopene and silymarin’s possible roles as herbal treatments against aflatoxicosis in Pekin duckling.

## Materials and Methods

### Ethical approval

All the applicable ethical guidelines for animal were followed during handling and sample collection from birds and adequate measures were taken to minimize pain or discomfort of selected birds were approved from Animal Health Research Institute (AHRI), Tanta Branch, Agriculture Research Centre, Giza, Egypt, with approved date AHRI 31/12/2019.

### Study period and location

The study was conducted during March and April 2020 at AHRI, Tanta Lab and Dokki lab.

### Ration content

The duckling’s rations were examined to detect the amount of AFs (AFs, ochratoxin and zeranol) using a basic methanolic extraction, monoclonal antibody immunoaffinity column clean-up, and a fluorometric detection method described by Scott and Kanhere [21], Trucksess *et al*. [22]. The chemical composition of the naturally basal ration constituted fat, protein, ash, fiber, moisture, carbohydrate, Ca, Pb, AFs (4%, 19.8%, 6.5%, 5.2%, 10%, 54.5%, 1.3%, and 0.4%), respectively, according to AOAC, 2000, methods, and 30 ppb for AFs were detected in the contaminated ration by fluorometers.

### Feed additives

Lycopene product 20 mg (LYC-O-MATO®) from natural tomato extract in the form of soft gel capsules, under Glen Ellyn license, Rd Bloomingdale, IL60108**,** USA. Silymarin 80% is a product of Chemical Industrial Development Company, Egypt. It was purchased under MDAAUS license, GmbH, Germany, with a dosage of 600 mg/kg body weight [23].

### Experimental design

We acquired one hundred and twenty one-day-old Pekin ducklings from a private farm and classified them into four groups, with 30 ducklings in each group. In the control group (G1), the ducklings were fed a ration free from AFs. In the second group (G2), the ducklings were fed a ration contaminated with 30 ppb of AFs. In the third group (G3), the ducklings were fed a ration contaminated with 30 ppb of AFs and treated with lycopene 100 mg/kg [24]. In the fourth group (G4), the ducklings were fed a ration contaminated with 30 ppb of AFs and treated with silymarin 600 mg/kg/ration [23]. After 2 weeks (the time by which signs of aflatoxicosis start to appear) of consuming the AFs contaminated ration, lycopene and silymarin were administered to treat aflatoxicosis for 10 days. The Pekin duckling received feed and water *ad libitum* and was subjected to 24 h light throughout the experiment under environmental conditions.

### Collection of blood and hepatic tissue samples

Throughout the experiment, blood samples were collected from the heart and placed for 30 min in a test tube for coagulation followed by centrifugation (3000 rpm for 15 min) and preserved at −20°C until use, measuring the liver and kidney enzymes. Immediately, the liver was removed and cleaned with sterile saline (0.9% NaCl). Samples were separated into two sections. One section was used to detect oxidant/antioxidant parameters, and the other was used for AFs residue detection.

### Detection of serum hepatic and kidney function

The following were detected to test for hepatic function, alanine aminotransferase (ALT) and aspartate aminotransferase (AST), alkaline phosphatase (ALP), glutamyl transferase (g-GT), albumin concentration, and total protein, following literature guidelines [25-29]. The kidney function was tested by detecting creatinine and uric acid following literature guidelines [30,31].

### Oxidant/antioxidant hemostasis in hepatic homogenates

We used phosphate-buffered saline (0.1 M, pH 7.4) to wash the liver tissue and homogenated it with cold potassium phosphate buffer (50 mM, pH 7.5) using a homogenizer with disposable tips. The prepared homogenate was centrifuged at 10,000×g at 4°C for 15 min. The resultant supernatant was taken to detect (malondialdehyde [MDA] and antioxidant biomarkers, including total antioxidant capacity (TAC), catalase (CAT) activity, and glutathione S-transferase (GST) following literature guidelines [32-35].

### Detection of AF residue in ration and liver

The quantitative determination of AFs in rations was done using the methanolic extraction method, monoclonal antibody immunoaffinity column clean-up. The fluorometer was used to determine a 30 ppb of AF in the ration according to Trucksess *et al*. [22]. AFs residue in the liver was estimated according to Jørgensen and Petersen [36].

### Statistical analysis

One-way analysis of variance analyzed the study data. The differences among the treated groups were found by Tukey’s HSD *post hoc* test using statistical version 22.0 of SPSS for Windows (IBM, Armonk, NY, USA). The study results are represented as the mean±standard error of means. The statistical level of significance was set at p<0.05 [37].

## Results

### Liver and kidney function

Table-1 presents the detrimental effect of aflatoxicosis and the potential therapeutic role of lycopene or silymarin on liver and kidney function biomarkers. The following ALT, AST, g-GT, and ALP enzyme activities were significantly elevated (287%, 186%, 396%, and 138%, respectively) in the serum of AFs intoxicated group. In comparison, they decreased significantly in the lycopene-treated group with 92%, 36%, 68%, and 20% and the silymarin group by 106%, 54%, 162%, and 10%. Furthermore, serum total protein and albumin were significantly reduced (14% and 12%) in the AFs intoxicated group and elevated in the lycopene-treated group (4.8% and 3.3%) and silymarin-treated group with 8% and 5%.

**Table-1:** Lycopene and/or silymarin effect on one-day-old Pekin duckling intoxicated with AF on ALT, AST, ALP, total protein, albumin, creatinine, and uric acid in comparison with control for 10 successive days n=10.

Liver and kidney enzymes	CTR	AFs	LYCO	SILYMA
ALT, μ/L	12.800±0.86^c^	049.50±1.75^a^	24.60±1.47^b^	26.40±1.12^b^
AST, μ/L	33.800±0.73^d^	096.75±1.77^a^	45.75±1.80^b^	52.00±0.95^c^
γ-GT, μ/L	12.400±0.81^d^	061.50±1.28^a^	20.80±0.58^c^	32.50±0.81^b^
ALP, μ/L	49.600±2.25^c^	118.25±2.69^a^	59.60±2.38^b^	54.80±1.39b^c^
Total protein, g/dL	07.620±0.08^a^	006.54±0.15^c^	07.26±0.06^b^	07.00±0.03^b^
Albumin, g/dL	03.620±0.04^a^	003.18±0.09^c^	03.50±0.03ab	03.44±0.02^b^
Creatinine, mg/dL	01.178±0.05^b^	001.46±0.06^a^	01.34±0.02^a^	01.32±0.04^a^
Uric acid, mg/dL	06.140±0.07^a^	006.24±0.07^a^	05.80±0.11^b^	06.26±0.04^a^

All values were expressed as mean±standard errors. All values indicated by different letters ^(a, b)^ are significantly different between groups within the same rows (p≤0.005). CTR=Control, AFs=Aflatoxins, LYCO=Lycopene, SILYMA=Silymarin, AF=Aflatoxins, ALT=Alanine aminotransferase, AST=Aspartate aminotransferase, ALP=Alkaline phosphatase

Creatinine and uric acid biomarkers of kidney function were measured, there was a significant increase in creatinine in AFs intoxicated group (20%), lycopene (14%), and silymarin (12%) as compared to the control group. There was no significant change in uric acid detected in all the groups.

### Oxidant/antioxidant parameters in hepatic tissue

**Figure-1:**
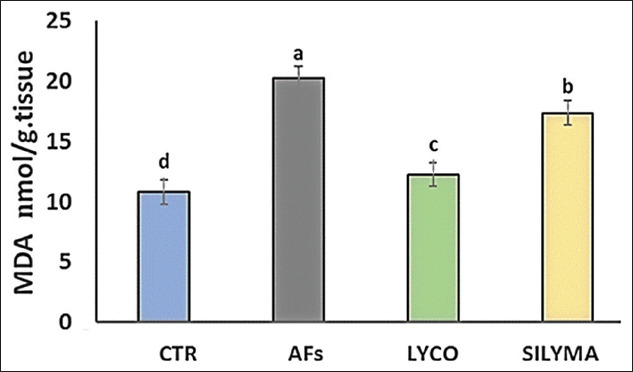
Hepatic lipid peroxidation parameters MDA, in control and treated groups in a one-day-old Pekin duckling. Different letters indicate significantly different mean values (p≤0.05),n=10. AF=Aflatoxin, LYCO=Lycopene, SILYMA=Silymarin, MDA=Malondialdehyde.

**Figure-2:**
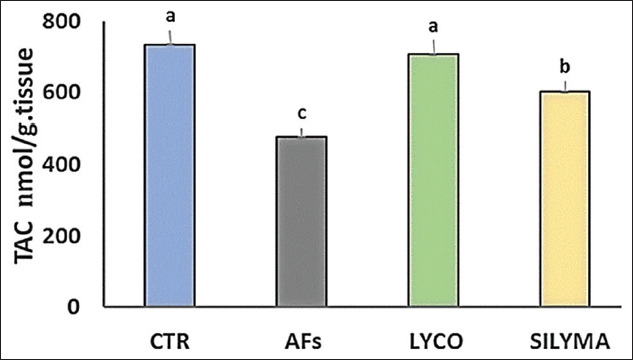
Hepatic TAC parameters in control and treated groups in a one-day-old Pekin duckling. Different letters indicate significantly different mean values (p≤0.05), n=10. AF=Aflatoxin, LYCO=Lycopene, SILYMA=Silymarin, TAC=Total antioxidant capacity.

**Figure-3:**
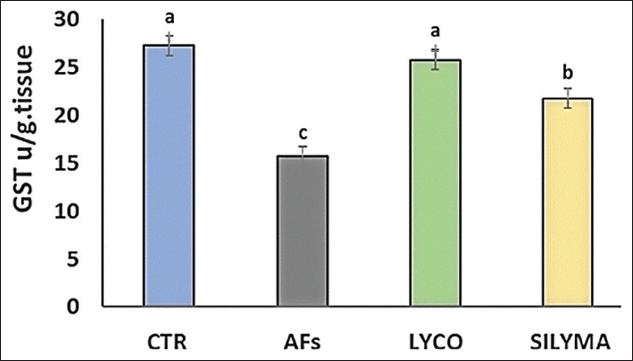
Hepatic GST parameters in control and treated groups in a one-day-old Pekin duckling. Different letters indicate significantly different mean values (p≤0.05), n=10. AF=Aflatoxin, LYCO=Lycopene, SILYMA=Silymarin, GST=Glutathione S-transferase.

**Figure-4:**
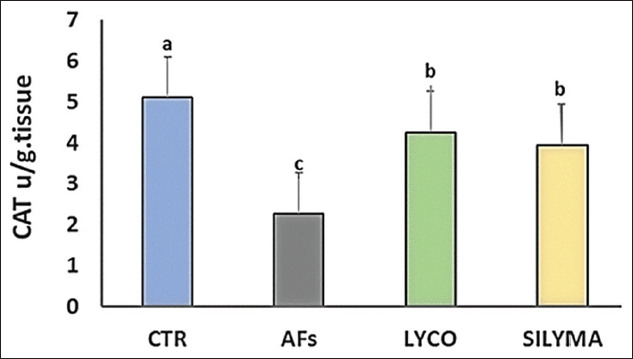
Hepatic CAT parameters in control and treated groups in a one-day-old Pekin duckling. Different letters indicate significantly different mean values (p≤0.05), n=10.AF=Aflatoxin, LYCO=Lycopene, SILYMA=Silymarin, CAT=Catalase.

Data analysis indicated a significant (p<0.05) elevation in MDA levels in the AFs intoxicated group and a significant reduction in the lycopene- and silymarin-treated groups compared to the control, as shown in Figure-1. The antioxidant parameter (TAC) was significantly decreased in AFs intoxicated group compared to the control and silymarin, while, in the lycopene-treated group, there was no significant effect, as shown in Figure-2. GST level was statistically reduced in the AFs intoxicated group compared to the control. However, the lycopene- and silymarin-treated groups showed elevated GST levels compared to the control, as shown in Figure-3. Furthermore, catalase activity was significantly reduced in the AFs intoxicated group, compared to the control, while it increased in the lycopene- and silymarin-treated groups, as shown in Figure-4.

### AFs residue in hepatic tissue

**Figure-5:**
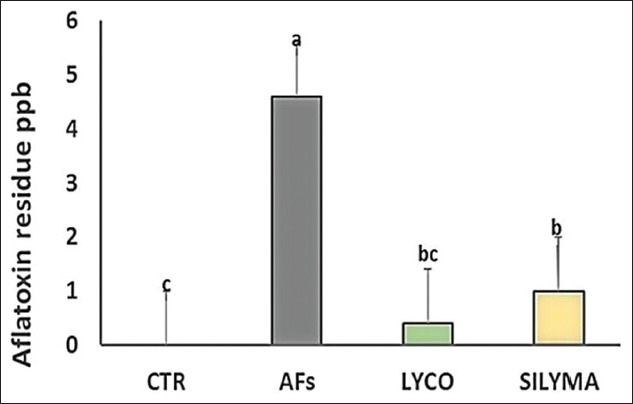
Hepatic AFs residue in intoxicated groups with AFs and intoxicated group and treated with LYCO or SILYMA in a one-day-old Pekin duckling. Different letters indicate significantly different mean values (p≤0.05), n=10. AF=Aflatoxin, LYCO=Lycopene, SILYMA=Silymarin.

A significant increase in AF residue in liver tissue was observed in the intoxicated group with AFs compared to the control group. While in the lycopene- and silymarin-treated group, AF residue was significantly reduced compared to the control, as shown in Figure-5.

## Discussion

The biochemistry results indicated elevated activity of AST, ALT, ALP, and g-GT in the AFs intoxicated group due to liver damage [38]. As a consequence of AFs intoxication in different livestock animals, the growth, immunity, egg production, and hepatic functions were adversely affected [39,40].This resulted from excess ROS production leading to oxidative damage and cellular membrane instability, facilitating the liver and kidney enzymes’ secretion into serum [38]. Furthermore, serum proteins, albumin, and total protein were significantly reduced in the AFs intoxicated group compared to the control due to hepatic damage resulting in impaired protein synthesis [41]. Overall, a rise in creatinine and uric acid level indicated that AFs exposure had a toxic and stressful effect on the kidney tissue, leading to degenerative kidney tissue changes and kidney dysfunction. The deleterious effects of AFs on the kidney are observed in the form of reduction of calcium, sodium, potassium, inorganic phosphate, and elevation of urea, creatinine, and uric acid levels, which are indices of kidney dysfunction as reported in this study [42]. The elevation of MDA resulted in a hepatic injury associated with the exhaustion of antioxidants status as GST, GPX, CAT, SOD, G6PD, and reduced glutathione [43,44]. MDA is a reactive aldehyde end product that has been elevated in the intoxicated group with AFs in this study. It may be due to lipid peroxidation with polyunsaturated fatty acid degradation in the cell membrane [45].

There was a significant reduction in MDA level in the lycopene-treated group with improvements in the antioxidant levels of TAC, CAT, and GSTs. The deleterious effect of AFs is directly linked to the bioactivation process and oxidative destruction in the liver, which is considered the key organ in humans and other animal models [46,47]. Lycopene may alleviate AF-induced hepatotoxicity and renal dysfunction through its beneficial properties [48,49]. Lycopene can overcome the elevated serum AST and ALT levels due to abnormality in hepatic parts and degeneration and necrosis of liver cells through the reducing amount of AFs metabolites or the induction of oxidative damage as documented in this study [50].

Using a silymarin extract led to significantly reduced ALT, AST, and γ-GT levels in intoxicated AFs group in comparison to the control group. This reduction might be due to the silymarin-mediated inhibition of toxins entering the cells and preventing the disturbances in hepatocyte membranes stabilization [51]. Furthermore, silymarin can maintain hepatocyte membrane integrity and reduce the entrance of toxic xenobiotics substances. Its phenolic nature is capable of donating electrons to stabilize free radicals and reactive oxygen species formation. It also affects the intracellular glutathione, which inhibits the lipoperoxidation of membranes [52] which may protect birds from the deleterious effect of AFs.

The public health hazard has warned that AFs residues in food and edible parts are life-threatening and maybe lethal [53]. AFs residue was reduced in the lycopene- and silymarin-treated groups. The presence of AFs residue in the lycopene group to match the control group may be because AFs have two metabolic pathways. Phase 1 involves the activation process blocked by lycopene. Phase 2 is activated by enzymes responsible for the detoxification process of AFs, consequently enhancing AFB-NAC production, resulting in lowering the level of AFs in the liver and urine [54].

## Conclusion

The current analysis was done on aflatoxicosis and the beneficial effects of lycopene and silymarin. ROS induction that led to the elevation of oxidative damage in hepatic tissue was discussed in this field case in Pekin duckling. AF-induced oxidative damage observed by changes in serum biochemical parameters, oxidant/antioxidant balance system, liver and kidney dysfunction, and accumulation in the liver cell. The consumption of AFs with lycopene or silymarin had a significant positive effect on the intoxicated group with AFs might be due to its antioxidant activity. It could be concluded that lycopene and silymarin herbal extracts are ecofriendly antioxidant compounds with a highly natural protective effect against AFs toxicity.

## Authors’ Contributions

SME designed the experiments, handled the animals, and analyzed the data. AFE and NAS contributed in handling the animals, sample collection, and contributed reagents/materials. DHS and SME designed the experiment and analyzed the data. DHS conceived and designed the experiments, analyzed the data, and reviewed draft of the paper. All authors have checked and approved the final version of the manuscript.
